# A comparative study regarding distance learning and the conventional face-to-face approach conducted problem-based learning tutorial during the COVID-19 pandemic

**DOI:** 10.1186/s12909-021-02575-1

**Published:** 2021-03-03

**Authors:** Chi-chung Foo, Billy Cheung, Kent-man Chu

**Affiliations:** 1 Department of Surgery, Li Ka Shing Faculty of Medicine, University of Hong Kong, Hong Kong, SAR China; 2 Department of Surgery, Queen Mary Hospital, Hong Kong, SAR China

**Keywords:** Problem-based learning, PBL, Distance learning, Online education

## Abstract

**Background:**

Educational pedagogies were modified during the COVID-19 pandemic to minimise interruption to teaching. One approach has been the distance learning problem-based learning (PBL) tutorial utilising the online peer-to-peer platform. The aim of this study was to compare the performance of students using distance learning PBL tutorials using with that of students utilising the conventional face-to-face approach.

**Methods:**

This retrospective study was conducted in a single academic institution. We compared two groups of fourth-year medical students from the same class: one group used distance learning (DL); the other, the face-to-face (FF) method. We used students’ baseline performance at the preceding block for one-to-one propensity score matching. Students utilising the PBL tutorial were given grades by their tutors according to a standardised scoring system encompassing five key areas (score range: 0–10). The main outcome was a student’s total score (i.e., the sum of the scores from the five key areas, ranging from 0 to 50).

**Result:**

We matched 62 students in each group. With four tutorials, there were 490 observations, with 245 in each group. The mean total score for the DL group was 37.5 ± 4.6, which was significantly lower than that of the FF group (39.0 ± 4.4, *p* < 0.001). We noted that students in the DL group had a significantly lower scores for all five areas of proficiency: participation, communication, preparation, critical thinking and group skills.

**Conclusion:**

Findings of this study revealed that the performance of students utilising the DL PBL tutorials was lower than that of students participating in the conventional FF approach. Further studies are needed to ascertain the underlying cause.

**Supplementary Information:**

The online version contains supplementary material available at 10.1186/s12909-021-02575-1.

## Background

During the first half of 2020, the world was challenged by the coronavirus pandemic on an unprecedented scale. In response, many people adopted the practice of social distancing, and schools suspended classes and activities. Medical students were devoid of opportunities to enter hospital premises because of tightened infection control measures. Educators adopted innovative measures to maintain learning opportunities for students who stayed at home [1–3]. Some of these measures, including online lectures or webinars, were in place before the COVID-19 outbreak [4]. Others were hastily put into place during the pandemic. Given its user-friendly design, online peer-to-peer platforms became extremely popular. Lectures, tutorials, skills demonstrations, and even bedside teaching for medical students can be conducted via this type of platform [5, 6]. For example, at the University of Hong Kong Li Ka Shing Faculty of Medicine offered a FF PBL tutorial using online peer-to-peer platform software. To many, such adaptations served as a lifeline to continue medical education during the coronavirus outbreak. It was also envisaged that some of these educational adaptations would persist after the pandemic. How effective these adaptations have been and how they compare with the conventional teaching method should be evaluated. A study on surgical skills teaching reported that using Web-based DL was well-received by undergraduate students [6]. The aim of this study was to evaluate the proficiencies in five key areas of students who took PBL tutorials by DL, an adaptation during the COVID-19 pandemic, and to compare them with the proficiency levels of students who learned via the conventional FF method.

## Methods

This was a retrospective study conducted in May 2020 at the Li Ka Shing Faculty of Medicine, University of Hong Kong; it was approved by the Institutional Review Board of the University of Hong Kong/Hospital Authority Hong Kong West Cluster (IRB reference number: UW 20–381). The subjects were medical students who were in their fourth year of their six-year medical curriculum. These students had been exposed to the PBL teaching approach since their first and second years and were familiar with the format. In their fourth year, students in this class were split into three groups, with each rotating through three Junior Clerkship (JC) rotation blocks-- Medicine, Surgery, and Multidisciplinary clerkship-- between November 2019 and April 2020. From February to May 2020, classes were suspended because of the outbreak of the severe acute respiratory syndrome coronavirus 2 (SARS-CoV-2). The conventional FF PBL in the Surgery block was replaced by DL, using the online peer-to-peer platform software ZOOM (Zoom Video Communications, San Jose, CA, USA). The tutors, content, group size, duration, and assessment criteria remained the same. All students from rotation one had participated in conventional PBL tutorials before the class suspension, whereas students from rotation three had engaged in DL (online) PBL exclusively after the outbreak.

Eight cases were presented for discussion in a total of four tutorials. We gave the paper-based case materials to students prior to the tutorials and encouraged pre-class preparation. The PBL scenarios included breast mass, neck swelling, rectal bleeding, abdominal distension, haematuria, acute retention of urine, abdominal pain in an adult patient and abdominal pain in a paediatric patient. Each tutorial lasted for two hours and was considered sufficient for students to go through two scenarios, discuss the relevant history and physical examination findings, decide on the suitable investigations, come up with working diagnosis and suggest the appropriate management. The group size was 11–12 students. Students were randomly allocated into groups; they remained in the same groups throughout the clerkship. Tutors were randomly assigned, and students had different tutors for the four tutorials. The scenarios were described over several pages and some leading questions were given. Students discussed approaches to the clinical problems and explored related issues. They addressed one or more learning objectives that were considered relevant. Tutors acted as facilitators and played minimal roles unless students strayed from a case. At the end of the session, tutors used a standardised form for evaluating the proficiency levels of students in five key areas: participation, communication, preparation, critical thinking and group skills. Tutors expected students to demonstrate adequate preparation on the applicable topic prior to each tutorial, active engagement in group discussions, adequate communication skills for expressing their viewpoints and raising relevant questions, the ability to manage controversies rationally, and attentiveness to other members without dominating the discussion. A score from 1 to 10 was given for each of these areas, with 10 being the highest. The total score represented the sum of the scores from all five key areas.

We compared the PBL performance of students in rotation three-- the DL group using the online platform – to that of students in rotation one, the conventional FF group; the latter functioned as the control group. We retrieved their PBL outcomes and overall assessments for the preceding Clinical Foundation Block (CFB), taken during the period August to October 2019, for baseline comparison. The CFB tutorials were all conducted using the conventional FF method; for these five PBL tutorials, students were assessed with the same evaluation form (scores ranging from 0 to 10). The overall assessment comprised the PBL assessment (20%), small group/bedside skills learning (60%), and a logbook (20%). Students in the FF group and DL groups were matched by propensity scores according to their performance (i.e., using PBL scores from the CFB). Matching was one to one, using the nearest neighbour method and tolerance of 0.5. Categorical variables were compared using the χ^2^ test. Continuous variables were compared with the independent sample *t*-test. A *p*-value of < 0.05 was considered statistically significant. The statistical analysis was performed using IBM SPSS version 25 (IBM, USA).

## Results

There were 77 and 75 students in the FF and DL groups, respectively. After propensity score matching, 62 students remained in each group. Matching for the remaining 15 and 13 students in the FF and DL groups, respectively, were not possible; therefore, they were excluded. Twenty-nine tutors were involved. With four tutorials, there were a total of 496 observations (248 per group). However, there were three absentees in the FF and DL groups, respectively, resulting in 245 observations per group. Gender composition, age, ethnicity and overall assessments for the CFB of the two groups are shown in Table 1, indicating comparability between the two groups. Their PBL performance in the preceding CFB was also comparable after propensity score matching (79.5 versus 79.9, *p* = 0.737).

**Table 1:** Demographics of the two groups

	FF group	DL group	
	*N* = 62	N = 62	p
Age (years)	21.8 ± 1.2	21.7 ± 1.0	^a^0.686
Gender			^b^0.368
Male	31 (50.0%)	36 (58.1%)	
Female	31 (50.0%)	26 (41.9%)	
Ethnicity			^b^0.619
Chinese	61 (98.4%)	59 (95.2%)	
Non-Chinese	1 (1.6%)	3 (4.8%)	
Overall performance at CFB (0–100)	77.3 ± 5.9	78.2 ± 4.6	^a^0.083
PBL performance at the CFB (0–100)	79.5 ± 9.3	79.9 ± 6.2	^a^0.737

*FF* Face-to-face

*DL* Distance learning

*CFB* Clinical Foundation Block (the preceding block)

The number inside the parenthesis indicated the range of score, with zero being the lowest

^a^comparison by independent sample *T* -test

^b^comparison by χ^2^ test

The PBL performance of the two groups during JC is shown in Table 2. Students in the FF group scored significantly higher. The mean total score for the DL group was 37.5, which was significantly lower than the score for the FF group (39.0, *p* < 0.001). Moreover, assessments regarding participation, communication, preparation, critical thinking and group skills were uniformly lower for the DL group compared to those for the control group.

**Table 2:** A comparison of PBL performance between the two groups

Total score^a^				
	FF group	DL group		
	*N* = 245	N = 245	^b^p	^c^Effect size
Participation (0–10)	7.9 ± 1.0	7.5 ± 1.0	< 0.001	0.40
Communication (0–10)	7.8 ± 0.9	7.6 ± 1.0	0.002	0.21
Preparation (0–10)	7.8 ± 0.9	7.5 ± 1.0	< 0.001	0.32
Critical thinking (0–10)	7.8 ± 0.9	7.5 ± 0.9	0.001	0.33
Group Skills (0–10)	7.8 ± 0.9	7.4 ± 1.0	< 0.001	0.42
Total score (0–50)	39.0 ± 4.4	37.5 ± 4.6	< 0.001	0.33

The number inside the parenthesis indicated the range of score, with zero being the lowest

*FF* Face-to-face

*DL* Distance learning

^a^mean ± standard deviation

^b^comparison by independent sample *T* -test

^c^Cohen’s d was used to represent the effect size

**Fig. 1:**
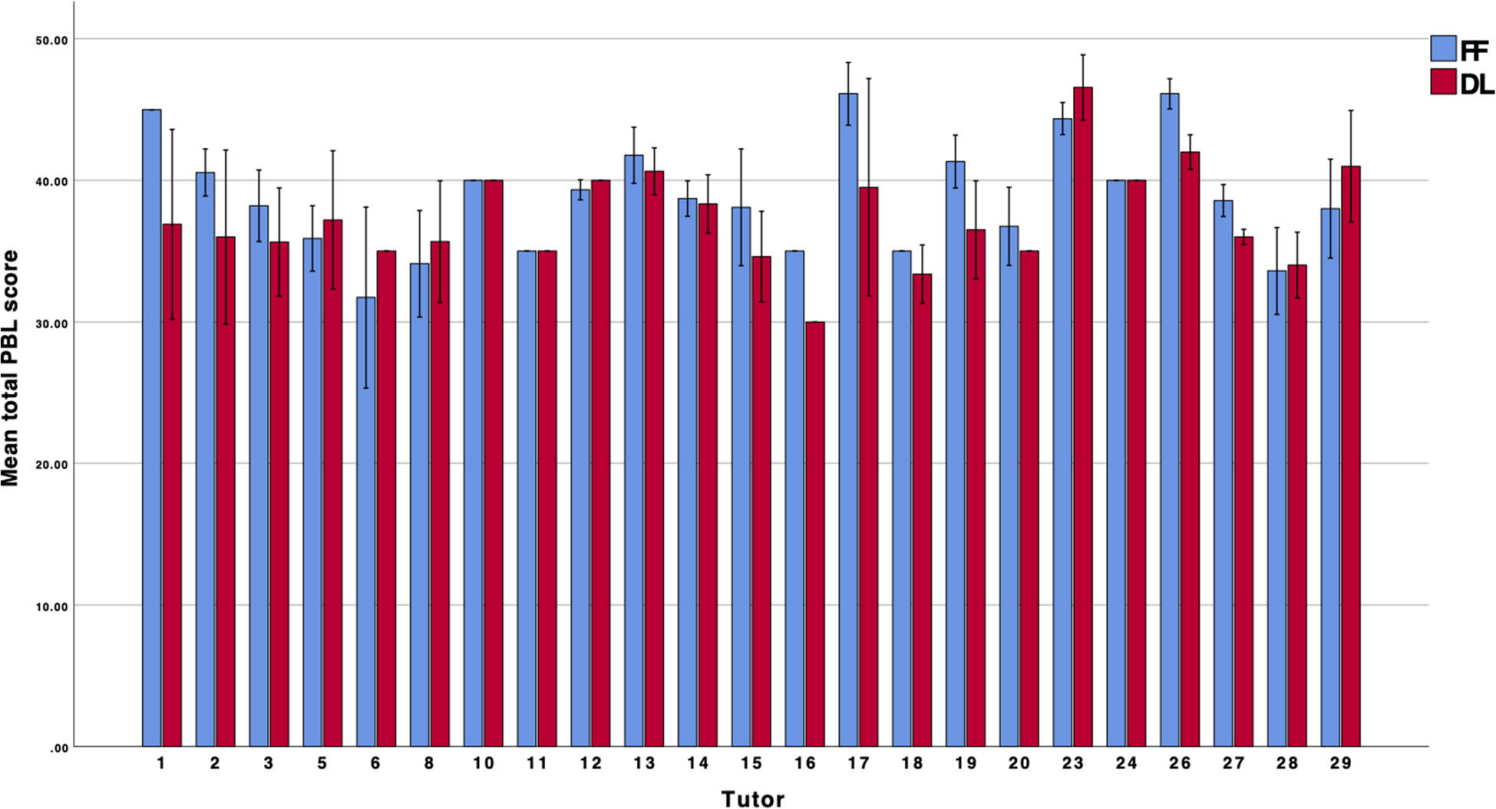
Mean PBL scores according to tutors

A subgroup analysis was performed to evaluate the effect of different tutorials and tutors. Table 3 shows a comparison of students’ performance for the four different tutorials. The mean total score was higher for the four tutorials; the difference was only significant for the first and third tutorials. A comparison of the two groups was also performed for individual tutors. Of the 29 tutors involved, six were excluded because they taught students in either the FF or DL group exclusively. Among the remaining 23, eight (34.8%) rated the proficiencies of students in the FF group higher and two (8.7%) rated those of students in the DL group higher (Fig. 1). The difference was not significant for remaining 13 tutors (56.5%).

**Table 3:** Subgroup analysis on the student performance at various tutorials

		Total score^b^		
Tutorial	Case	FF	DL	^a^p
1	1 and 2	40.4 ± 3.2	38.9 ± 3.3	0.012
2	3 and 4	40.6 ± 5.0	39.2 ± 5.5	0.146
3	5 and 6	37.6 ± 4.1	34.9 ± 4.0	< 0.001
4	7 and 8	37.5 ± 4.1	36.8 ± 4.2	0.343

^a^comparison by independent sample *T* -test

^b^mean ± standard deviation

## Discussion

E-learning has been in place for some time [4]. Many have viewed it as the preferred mode of teaching for the future, as students are given more flexibility [7, 8]. This type of learning has become indispensable during the COVID-19 pandemic when social contact is minimal. However, e-learning has certain limitations [9]. It is reasonable to believe that many educational adaptations adopted during the pandemic will persist. Indeed some of the novel ones may result in a better overall learning experience for students. Therefore, it is worthwhile to evaluate them.

PBL was first popularised at the McMaster University in Canada [10, 11]. Contrary to traditional lecture-based teaching, PBL encourages active and student-directed learning. Students are trained in independent learning, teamwork, and communication skills [12]. Some have suggested that students who utilised PBL curricula have emerged as better problem solvers [13]. For a PBL tutorial group to be efficient, members’ initiation is crucial, with all striving to function as a productive members.

Findings of this study revealed that students using DL method performed at a significantly lower level than students learning via the conventional FF approach. One possible explanation was that students and tutors had to adapt a new way of conducting the PBL tutorial. Wilcha cited technical challenges like establishing a reliable internet connection, problems with hardware and software learning platforms, etc. as some of the weakness of online teaching in a systematic review [9]. However, the software was relatively user-friendly, and the format of the tutorials remained the same. The time needed for students and tutors to become familiar with the new ‘environment’ should have been minimal. Technical issues such as Internet connectivity and lag time did not seem to be major problems in this locality. The fact that lower performance was also observed at the third tutorial suggested there was more than a transitional issue.

Modern digital communication technology has allowed us to trump geographical barriers [14, 15]. Online platforms provide opportunities to meet and discuss without being physically close to each other. However, this type of technology may not reproduce the same interpersonal distance as physical presence [16]. Students may feel distant and detached from the rest of the group despite being connected via the computer screen and audio. The perception of being an outsider may reduce one’s eagerness to participate and contribute. In this study, students were required to keep the audio and video on throughout the tutorials, but there were occasions in which students only revealed or unmuted themselves when they were prompted to do so. Most students participated in the PBL tutorials from their residences via video conferencing. The casual ambiance might have appeared ‘unreal’ for learning, requiring psychological adaptation. Students were also more prone to distractions from surrounding persons or events. Prior studies have shown that DL using online platforms is associated with reduced student engagement, reduced communication and poor motivation [17–19].

Tutors can be affected too. Although tutors played minimal roles in this study, apart from evaluating students, they might have been inclined to intervene when needed and prone to be distracted. Nevertheless, these are only postulations; further research is warranted. A survey should be conducted to ascertain the perceptions of students and tutors regarding online tutorials and ways to improve the overall learning and teaching experience.

There were several limitations to this study. There was no randomisation, and the comparison was subjected to bias. The chance of bias was minimised by matching student performance at baseline. The tutor effect was another confounding factor. Although we used a structured evaluation form with clear guidance regarding scoring, there was a possibility of variations among tutors, with some being more stringent than others. Tutors in this study were regularly involved in PBL teaching, but there was no prior training or standardisation in terms of scoring. For some tutors, there was little variation in scores between the five areas of proficiency, which indicated that the tutors were more inclined to give an overall impression of students’ performance. This situation limited the ability to single out specific areas. There were tutors (tutors 10, 11 and 24 in Fig. 1) that gave every students the same score. Again this reduced the sensitivity to detect a difference, if any, between the two groups. It was postulated that this was why a lower score was observed in the DL group in tutorial two and four but the difference was insignificant. Additionally, tutorials for the two groups were conducted at different times, and students in the DL group were learning during a pandemic, which was clearly a torment to some. Thus, the negative psychological impact on them might have affected their performance. Furthermore, some classes or bedside teachings were suspended at the time. It has had been a suggested that people working from home during the pandemic may be more prone to loneliness, and hence, decreased efficiency [20].

## Conclusion

Innovative educational adaptations have been essential during the COVID-19 pandemic. However, further evaluation before permanent adoption is warranted. A direct transition from the conventional way of teaching into an online-based format may not have the same impact. This study showed that students who used DL PBL tutorials exhivited lower levels of proficiency in key area than students who utilised the conventional FF approach. Further studies are needed to ascertain the underlying cause.

## Supplementary Information

**Additional file 1.** The standardised form for tutors to evaluate students’ proficiency levels was attached as supplementary material.

## Data Availability

The datasets generated and / or analysed during the current study are available from the corresponding author on request.

## References

[1] Eva KW, Anderson MB. Medical education adaptations: really good stuff for educational transition during a pandemic. Med Educ. 2020;54(6):494. 10.1111/medu.14172 32233098

[2] Chick RC, Clifton GT, Peace KM, Propper BW, Hale DF, Alseidi AA, Vreeland TJ. Using technology to maintain the education of residents during the COVID-19 pandemic. J Surg Educ. 2020;77(4):729–32. 10.1016/j.jsurg.2020.03.018 PMC7270491 32253133

[3] Liang ZC, Ooi SBS, Wang W. Pandemics and their impact on medical training: lessons from Singapore. Acad Med. 2020;95(9):1359–61. 10.1097/ACM.0000000000003441 PMC7188065 32304387

[4] Kim S (2006). The future of E-learning in medical education: current trend and future opportunity. J Educ Eval Health Prof.

[5] Tsang ACO, Lee PP, Chen JY, Leung GKK. From bedside to Webside: a neurological clinical teaching experience. Med Educ. 2020;54(7):660. 10.1111/medu.14175 PMC7262309 32285492

[6] Co M, Chu KM. Distant surgical teaching during COVID-19 - a pilot study on final year medical students. Surg Pract. 2020. 10.1111/1744-1633. 10.1111/1744-1633.12436 PMC7361811 32837531

[7] Shah D (2016). Online education: should we take it seriously?. Climacteric.

[8] Ruiz JG, Mintzer MJ, Leipzig RM (2006). The impact of E-learning in medical education. Acad Med.

[9] Wilcha RJ (2020). Effectiveness of virtual medical teaching during the COVID-19 crisis: systematic review. JMIR Med Educ.

[10] Neufeld VR, Barrows HS (1974). The "McMaster philosophy": an approach to medical education. J Med Educ.

[11] Neville AJ, Norman GR (2007). PBL in the undergraduate MD program at McMaster University: three iterations in three decades. Acad Med.

[12] Kamin CSDR, Wilson B, Armacost M, Breedon T (1999). The development of a collaborative distance learning program to facilitate pediatric problem-based learning. Med Educ Online.

[13] Distlehorst LH, Dawson E, Robbs RS, Barrows HS (2005). Problem-based learning outcomes: the glass half-full. Acad Med.

[14] Grange ES, Neil EJ, Stoffel M, Singh AP, Tseng E, Resco-Summers K, Fellner BJ, Lynch JB, Mathias PC, Mauritz-Miller K (2020). Responding to COVID-19: the UW medicine information technology services experience. Appl Clin Inform.

[15] Lamba P (2011). Teleconferencing in medical education: a useful tool. Australas Med J.

[16] Norman ETH, Huegel D (2016). The distance between us: using construal level theory to understand interpersonal distance in a digital age. Front Digit Humanit.

[17] Lee ICJ, Koh H, Lai SH, Hwang NC (2020). Academic coaching of medical students during the COVID-19 pandemic. Med Educ.

[18] Longhurst GJ, Stone DM, Dulohery K, Scully D, Campbell T, Smith CF (2020). Strength, weakness, opportunity, threat (SWOT) analysis of the adaptations to anatomical education in the United Kingdom and Republic of Ireland in response to the Covid-19 pandemic. Anat Sci Educ.

[19] Kaup S, Jain R, Shivalli S, Pandey S, Kaup S (2020). Sustaining academics during COVID-19 pandemic: the role of online teaching-learning. Indian J Ophthalmol.

[20] Loneliness: The other side of working from home https://www.medicaldaily.com/loneliness-other-side-working-home-working-home-lonely-lifestyle-workplace-452535. Accessed 1 June 2020.

