# Supplementary material for: A comparative study regarding distance learning and the conventional face-to-face approach conducted problem-based learning tutorial during the COVID-19 pandemic
Source: BMC Med Educ. 2021 Mar 3;21:141. doi: 10.1186/s12909-021-02575-1 (PMC7928185; doi:10.1186/s12909-021-02575-1)
Supplement: Supplementary file 1 — Additional file 1. The standardised form for tutors to evaluate students’ proficiency levels was attached as supplementary material. [file 12909_2021_2575_MOESM1_ESM.docx]

THE UNIVERSITY OF HONG KONG

M.112/805

LKS FACULTY OF MEDICINE

# **Assessment of Individual Student’s Performance at PBL Tutorials**

| Name of Student: |  | Group No: |  |
| --- | --- | --- | --- |
|  |  |  |  |
|  |  |  |  |
| Tutor: |  | System / Block: | Junior Clerkship Block B |

*(Please write all tutors’ name if assessed by more than one tutor)*

### Section I

Please **circle** your rating of the student in each of the following 5 categories:

## Participation

| **Poor** | | | **Unsatisfactory** | | | | **Average** | | **Above Average** | | **Excellent** | |
| --- | --- | --- | --- | --- | --- | --- | --- | --- | --- | --- | --- | --- |
| Little evidence of participation or interest. | | | Occasionally participates but usually at a superficial level. Shows limited interest. | | | | Makes some contributions, and sometimes suggests learning goals. Shows some interest. | | Good participation, and usually shows a deep understanding of the topic. Often suggests hypotheses and learning goals. | | Frequent and constructive participation. Always shows a deep understanding. Interested and enthusiastic. | |
| 1 | 2 | 3 | | 4 | 5 | 6 | | 7 | | 8 | 9 | 10 |
| Observation/ Comment: | | | | | | | | | | | | |

## Communication

| **Poor** | | | **Unsatisfactory** | | | | **Average** | | **Above Average** | | **Excellent** | |
| --- | --- | --- | --- | --- | --- | --- | --- | --- | --- | --- | --- | --- |
| Poor communication skills. Is unable to express simple ideas | | | Limited skills. Can express simple concrete ideas. | | | | Appropriate communication skills, but has some difficulty with abstract concepts. Tends to read directly from text. | | Good skills. Can express complicated ideas using simple language. | | Excellent skills. Always able to explain points and views clearly and precisely. | |
| 1 | 2 | 3 | | 4 | 5 | 6 | | 7 | | 8 | 9 | 10 |
| Observation/ Comment: | | | | | | | | | | | | |

## Preparation

| **Poor** | | | **Unsatisfactory** | | | | **Average** | | **Above Average** | | **Excellent** | |
| --- | --- | --- | --- | --- | --- | --- | --- | --- | --- | --- | --- | --- |
| Little evidence of preparation for tutorials. | | | Some preparation, but usually limited to single source, eg. textbook or lecture notes. | | | | Sometimes prepares well and uses more than one source, but synthesis sometimes uneven. | | Usually well prepared. Uses different sources and shows ability to synthesize different perspectives. | | Always well prepared, with deep understanding of material from multiple and locally relevant sources. | |
| 1 | 2 | 3 | | 4 | 5 | 6 | | 7 | | 8 | 9 | 10 |
| Observation/ Comment: | | | | | | | | | | | | |

## Critical Thinking

| **Poor** | | | **Unsatisfactory** | | | | **Average** | | **Above Average** | | **Excellent** | |
| --- | --- | --- | --- | --- | --- | --- | --- | --- | --- | --- | --- | --- |
| Does not question or challenge others. Does not recognize any errors nor raise any controversies. | | | Occasionally questions or challenges others. Shows a limited recognition of errors or controversial issues. | | | | Sometimes willing both to challenge others, and to respond to challenges. | | Often raises questions that display reflective thinking. Often points out problems or controversies during discussion and pursues further understanding. | | Can always discuss controversies with reasoning and data. Frequently asks questions that help promote a deeper understanding of the subject. | |
| 1 | 2 | 3 | | 4 | 5 | 6 | | 7 | | 8 | 9 | 10 |
| Observation/ Comment: | | | | | | | | | | | | |

## Group Skills

| **Poor** | | | **Unsatisfactory** | | | | **Average** | | **Above Average** | | **Excellent** | |
| --- | --- | --- | --- | --- | --- | --- | --- | --- | --- | --- | --- | --- |
| No apparent idea of group process. Uninvolved. | | | Passive, with limited group skills. Defensive and resistant when prompted. | | | | Adequate skills. Sometimes a passive member, but responds to prompting. | | Good group skills, and contributes actively to group activities. Sometimes helps others. | | Excellent group skills. Always attentive and encourages participation by others, but does not dominate the discussion. | |
| 1 | 2 | 3 | | 4 | 5 | 6 | | 7 | | 8 | 9 | 10 |
| Observation/ Comment: | | | | | | | | | | | | |

#### Section II

Please give your overall comments on the student in the space provided below. If you have given any rating below 6 or above 9 in any of the 5 categories, please elaborate on your assessment.

|  |
| --- |

Note: For cases of absence without any reason or justification, a progressive mark deduction system on the assessment will be imposed, e.g. 10% of the overall marks will be deducted for absence from one tutorial, 20% mark deduction for absence from two sessions, and so on and no mark will be given in case of absence from more than 50% of the tutorials. Upon receipt of this completed assessment form, the Faculty Office will, taking into account of the attendance record, finalize the marks for each individual student.

| SIGNED by: |  | (Tutor) |  | Date: |  |
| --- | --- | --- | --- | --- | --- |

August 2005
